# Design and implementation of 3-D printed radiation shields for environmental sensors

**DOI:** 10.1016/j.ohx.2022.e00267

**Published:** 2022-01-29

**Authors:** J.S. Botero-Valencia, M. Mejia-Herrera, Joshua M. Pearce

**Affiliations:** a Grupo de Sistemas de Control y Robótica, Instituto Tecnológico Metropolitano, Medellín, Colombia; b Department of Electrical & Computer Engineering, Ivey Business School, Western University, London, ON, Canada

**Keywords:** Climatic variables, Environmental variables, Internet of Things (IoT), low cost, Radiation shield, 3-D printing, Open hardware, Environmental monitoring, Sensing, Environmental sensing, Additive manufacturing, Smart agriculture

## Abstract

The measurement of outdoor environmental and climatic variables is needed for many applications such as precision agriculture, environmental pollution monitoring, and the study of ecosystems. Some sensors deployed for these purposes such as temperature, relative humidity, atmospheric pressure, and carbon dioxide sensors require protection from climate factors to avoid bias. Radiation shields hold and protect sensors to avoid this bias, but commercial systems are limited, often expensive, and difficult to implement in low-cost contexts or large deployments for collaborative sensing. To overcome these challenges, this work presents an open source, easily adapted and customized design of a radiation shield. The device can be fabricated with inexpensive off-the-shelf parts and 3-D printed components and can be adapted to protect and isolate different types of sensors. Two material approaches are tested here: polylactic acid (PLA), the most common 3-D printing filament, and acrylonitrile styrene acrylate (ASA), which is known to offer better resistance against UV radiation, greater hardness, and generally higher resistance to degradation. To validate the designs, the two prototypes were installed on a custom outdoor meteorological system and temperature and humidity measurements were made in several locations for one month and compared against a proprietary system and a system with no shield. The 3-D printed materials were also both tested multiple times for one month for UV stability of their mechanical properties, their optical transmission and deformation under outdoor high-heat conditions. The results showed that ASA is the preferred material for this design and that the open source radiation shield could match the performance of proprietary systems. The open source system can be constructed for about nine US dollars, which enables mass development of flexible weather stations for monitoring needed in smart agriculture.

## Graphical abstract

**Figure:**
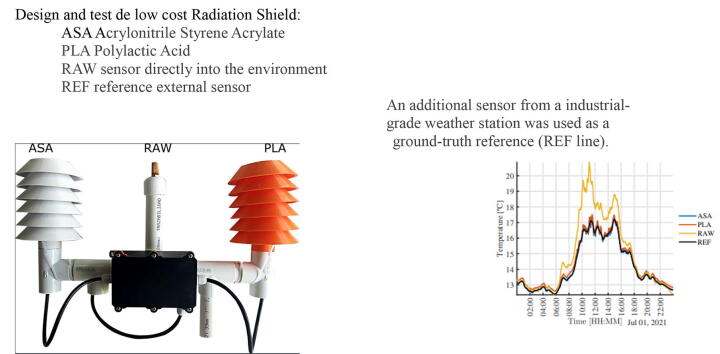

**Specifications table:**

**Table:** 

**Hardware name**	Open source radiation shield	
**Subject area**	• Engineering • Instrumentation • Internet of things	
**Hardware type**	• Measuring physical properties and in-lab sensors • Field measurements and sensors • Electrical engineering and computer science	
**Open source license**	GNU GPL v3 for documents and CERN OHL v2 for hardware	
**Cost of hardware**	5.70–9.00 USD	
**Source file repository**	https://doi.org/10.17605/OSF.IO/XE49Y	

## Hardware in context

The study of the environment is of interest in numerous different fields such as aviation, agronomy, hydrology, meteorology, biology, construction, energy and many others. Such studies often involve variables such as wind speed and direction, humidity, temperature, precipitation, etc. [1], [2], [3], [4]. Although some of these sensors are inexpensive and do not require strict protection against climate factors, other sensors such as humidity or temperature sensors require a higher degree of protection or encapsulation as poor outdoor deployment architectures can generate micro-climates with high humidity or temperature levels and consequently introduce a bias to the data provided by the monitoring system [5], [3], [6]. Thus protection devices against solar radiation and moisture are needed and usually designed with materials and shapes that reject UV radiation and allow air circulation at ambient temperature [7]. Although it is possible to find commercial enclosures for sensors that fulfill such tasks, they are often fragile and difficult to implement in low-cost contexts or large deployments of collaborative sensing. Many commercial devices are expensive [8], [9], [10], [11], which limits their accessibility in low-resource settings.In addition, they are only manufactured by a few companies making them difficult to source nationally to avoid costly import fees or tariffs in some countries.

Systems designed to hold and keep sensors protected are regularly known as radiation shields, although their function also includes humidity and temperature bias reduction [6]. These shields are often a stack of conical-shaped and flat-tipped plates with a specific separation (although it is also possible to find other geometric designs as a vented cylinder or inverted u-shaped systems) [4]. The cone or body material provides a UV shield that protects the sensors from solar radiation, and the separation between the plates or the perforations of the body allows airflow to decrease the accumulation of humidity and heat [7]. Seeking to reduce the manufacturing price, some systems have been developed using alternative materials with UV coatings or insulating layers [12], [4], [13], [7], [14]. In addition, there is now widespread agreement in the literature that the open source scientific hardware is less expensive than proprietary hardware [15], [16], [17], [18]. To leverage the distributed manufacturing paradigm to make open source digitally-replicable tools more accessible [19], this study presents a design of the cone stack setup shields, using a low-cost open source RepRap-class 3-D printer to make the core components [20], [21] and commercially available materials to assemble them. Two materials are tested. First, polylactic acid (PLA) is selected as it is a green biomaterial and the most widespread, accessible and generally low-cost 3-D printing filament with mechanical properties that can be controlled by accessible process parameters [22]. PLA is compostable under specific conditions [23] and can be considered biodegradable as it degrades via both UV and thermal mechanisms [24] and is thus known to become brittle when exposed to sunlight [25]. Second, acrylonitrile styrene acrylate (ASA) was tested as it provides better resistance against UV radiation, greater hardness, and generally higher resistance to degradation [26] compared to more common fused filament fabrication(FFF)-based materials such as PLA or acrylonitrile butadiene styrene (ABS) [27].

To validate the designs, the two prototypes (one of PLA and one of ASA) were installed on a custom outdoor meteorological system and temperature and humidity measurements were made in several locations for one month and compared against a proprietary system and a system with no shield. The 3-D printed materials were also both tested multiple times for one month for UV stability of their mechanical properties, their optical transmission and deformation under outdoor high heat conditions.

## Hardware description

The proposed device is primarily designed to protect humidity and temperature sensors and obtain non-biased measurements due to humidity concentration or lack of ventilation leading to high system temperatures. The system, also serves as a protective housing that can be used with other types of sensors. It is designed using an open source design methodology [28], [29], which maximizes the components that can be digitally replicated along with the use of off-the-shelf parts that are easily sourced in most places in the world. The shield is composed of six truncated cones, five of them with a central perforation, and one without a perforation that is located at the top of the system and acts as the roof of the system. The cones are arranged on each other using 20 mm metallic M3 separators, which allow airflow and keep the interior dry. A part designed for the sensor holder is added in the second cone (bottom to top) and secured with M3 screws, which ensures that the sensor does not receive direct light and is protected from the outside. Finally, a 1-inch PVC pipe coupling is added to the base of the system, which makes it easy to assemble in various configurations or locations. Since the parts are available in both STEP and STL format, it is possible to print them using different 3-D printing technologies, however, in this article, two versions were fabricated to compare the quality of the measurements acquired with the humidity and temperature Sensor SHT10, one in PLA material which is biodegradable and another version in ASA which provides better UV protection against solar radiation.

• The cost of the shield is much lower than commercial systems, approximately 25% of the value of the least expensive found on the market.
• It can be easily customized, replicated and experimented with other materials that further reduce the cost of production.
• System construction is modular, which makes it easy to incorporate more cones and adapt to different sensors without having to make major modifications.
• Provides a level of protection equal to that of commercially used systems, and is easy to assemble and install.

## Design files

### Design Files Summary

This section describes each of the files necessary for the construction of this device. The Table below also indicates its location.

**Fig. 1:**
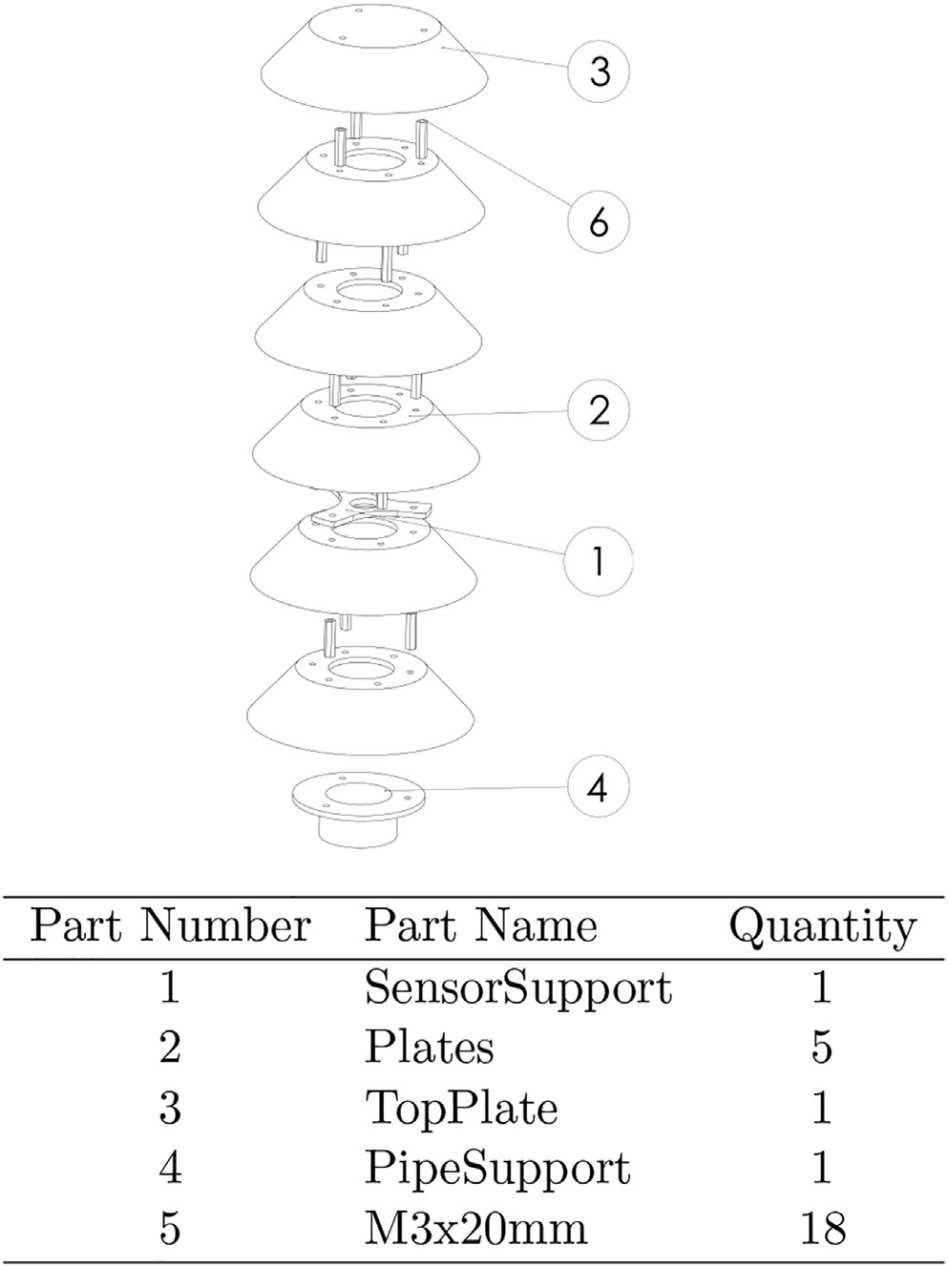
Exploded Assembly of the designed radiation Shield.
• The plates or cones (2 in the Fig. 1) are the intermediate pieces of the structure and directly protect the sensor. The sensor size determines the number of required parts.
• SensorSupport (1 in the Fig. 1), is a piece to set the sensor (SHT10) in the center of the structure, the internal diameter can be adjusted to fix another type of sensor.
• TopPlate (3 in the Fig. 1), is the top plate, the only difference with the other cones is that TopPlate does not have the central hole and only has three holes to fix.
• MainCode is the main source code used to read the sensors (SHT10), and send the data to the cloud. It has adjustable parameters.
• Schematic is a drawing where the electrical connections are shown in a simplified way. It includes the connections for the sensors.

• PipeSupport (4 in the Fig. 1) is a piece that can be 3-D printed and is used in the lower part of the assembly to join the plate structure to a 1-inch PVC tube.

**Table:** 

**Design file name**	**File type**	**Open source license**	**Location of the file**
PipeSupport	stl	GNU GPL v3.	https://osf.io/97mw5/
PipeSupport	step	GNU GPL v3.	https://osf.io/b5ax9/
Plates	stl	GNU GPL v3.	https://osf.io/bdxt2/
Plates	step	GNU GPL v3.	https://osf.io/w289y/
SensorSupport	stl	GNU GPL v3.	https://osf.io/gqnja/
SensorSupport	step	GNU GPL v3.	https://osf.io/5duw3/
TopPlate	stl	GNU GPL v3.	https://osf.io/bqj8u/
TopPlate	step	GNU GPL v3.	https://osf.io/gxzk9/
Assembly	pdf	GNU GPL v3.	https://osf.io/xrzqk/
MainCode	ino	GNU GPL v3.	https://osf.io/yf7xa/
Schematic	pdf	GNU GPL v3.	https://osf.io/62d9v/

## Bill of materials

This section presents a complete list of the parts that must be purchased and fabricated for the manufacture of this device. The BOM includes the current costs and an example supplier. The total cost shown enables researchers to carry out all of the tests discussed here, however, the cost of an open source radiation shield would be only 9.00 USD for ASA and 5.70 USD for PLA, if commercial filament is used.

**Table:** 

**Designator**	**Component**	**Qty**	**Unit cost**	**Total cost**	**Source of material**
Argon WiFi	MCU	1	$27.92	$27.92	t.ly/olZ0
Terminal Block	Base MCU	1	$14.95	$14.95	t.ly/GrLp
SHT10	Sensor	3	$26.90	$80.70	t.ly/iiL5
LiPo 6Ah	Battery	1	$29.95	$29.95	t.ly/LsLW
Box IP69	Structural	1	$12.94	$12.94	t.ly/x3f6
ASA filament	Structural	0.3	$29.99	$9.00	t.ly/9cQJ
PLA filament	Structural	0.3	$18.99	$5.70	t.ly/CBQr
Enclosure Gland PG7	Structural	1	$8.99	$8.99	t.ly/HOL8
Pipe PVC 1-in	Structural	1	$3.79	$3.79	t.ly/nj0q
Tee PVC 1-in	Structural	5	$1.41	$7.05	t.ly/Bo7K

				$200.98	Total

## Build instructions

The open source sensor shield system was developed in a modular way. This enables users to add different numbers of components to fit several sensors. Additionally, the design enables users to test the effect of various configurations such as a changing the number of cones, materials, or cone separations in various environments. The tested system consists of six truncated cones with 20 mm of separation arranged as seen in the Fig. 1. Initially, the cone without central perforation must be joined with one of the perforated cones using M3 separators of 20 mm height and screws of the same type as seen in the Fig. 1. Each cone has six M3 perforations where screws must be inserted leaving an intermediate hole and fixed with the separators to join with the other cones. The lower cone must be fixed to piece number 5 that helps to introduce a 1-inch PVC tube, while piece number 1 is attached to the second cone (bottom to top) using M3 perforations. Finally, the central hole of the cones allows air flow and serves as a conduit for installing sensors and for wiring them.

## Operation instructions

**Fig. 2:**
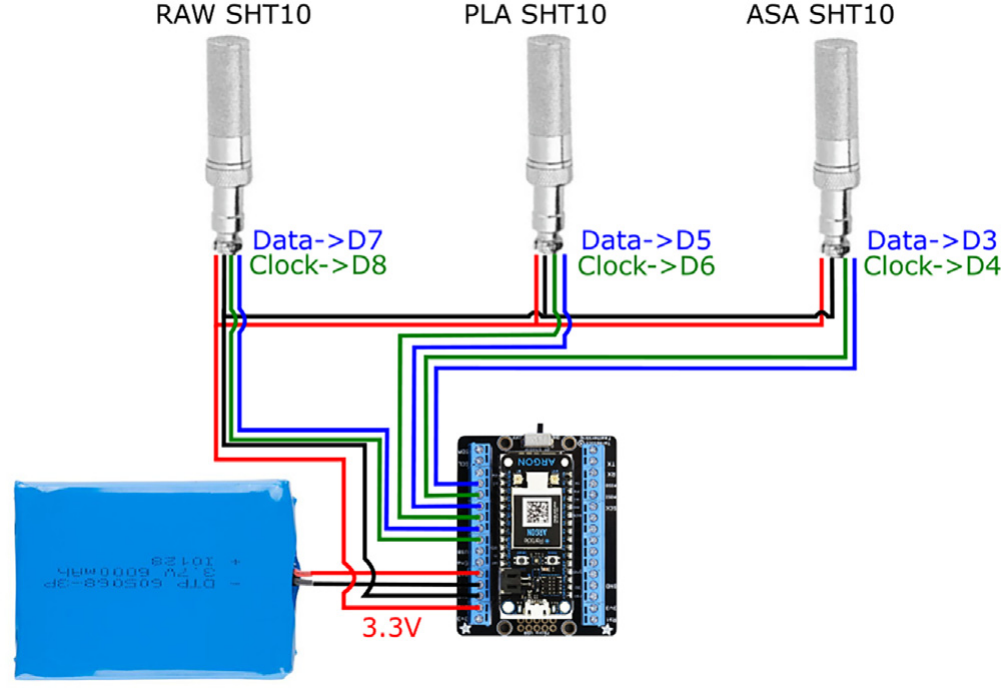
Connection diagram for the validation electronic system..

The design presented in this work only requires that it is assembled taking into account the instructions in the previous section in order to operate it normally. Here, in order to test different materials to make a comparison, the assembly presented in the Fig. 2 can be used, and the source code was included in the Open Science Framework repository for the project. This code acquires the output from the three sensors and sends the data to the cloud using MQTT in real time.

## Validation and characterization

**Fig. 3:**
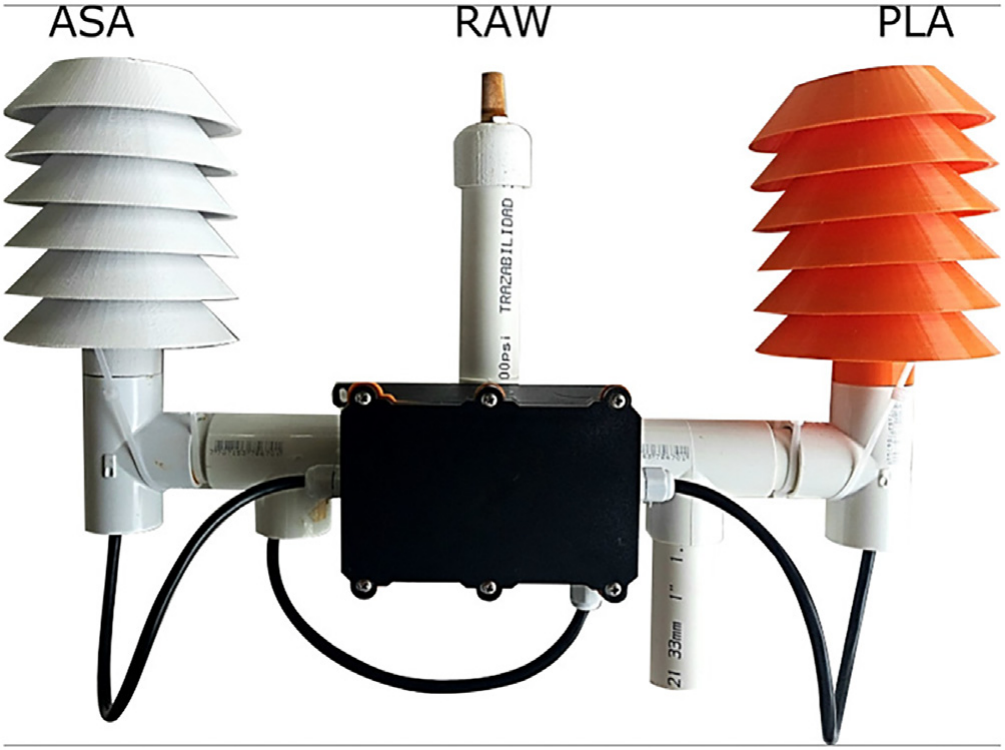
Assembly used for validation and testing.

**Fig. 4:**
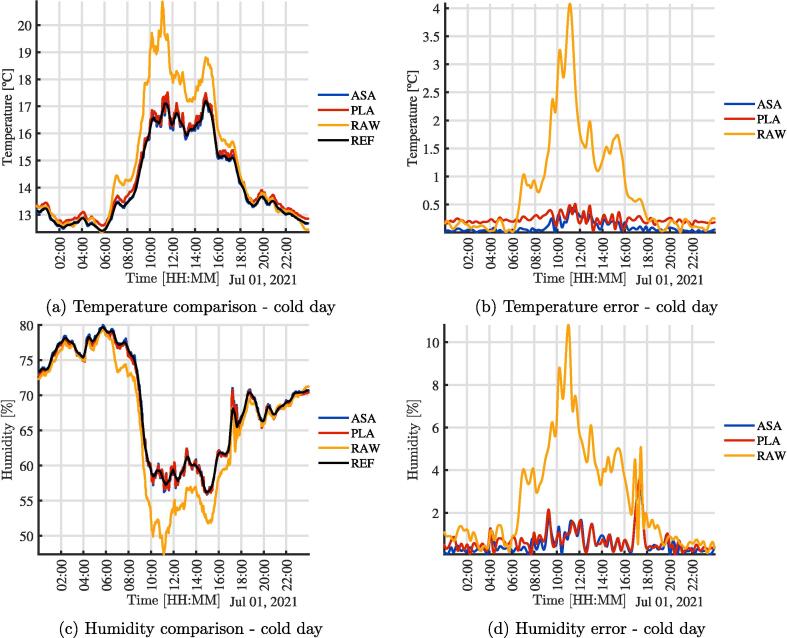
Comparison of temperature and humidity, Bogotá, day 1, cold day.

To test the effect of the radiation shield, three SHT10 temperature, and humidity sensors were mounted as shown in Fig. 2. An additional sensor from a Vantage Pro station was used as a ground-truth reference [30]. Fig. 2 shows the wiring between the electronic elements of the sensors for the comparison experiment presented in this work. Three sensors were used to have: 1) a sensor exposed to the environment, 2) a sensor with the radiation shield fabricated in PLA and 3) a second one made from ASA. In the figures ”REF” is the Vantage Pro sensor, ”ASA” the sensor built with the ASA shield, ”PLA” corresponds to the sensor with the shield made of PLA, and ”RAW” the sensor exposed to the environment without a shield. In Fig. 3, the assembly used to validate and test the operation of the sensors is presented. It can be similarly integrated into open source climate stations [31]. A radiation shield is 3-D printed in PLA (Orange) and one in ASA (White). In addition, a sensor is directly exposed to the environment. All three were joined mechanically with a structure fabricated in 1-inch PVC tubes and connected in the central black box as seen in Fig. 3. The microcontroller is responsible for acquiring and sending data to the cloud for analysis. The sensor used is the same in all three cases, the SHT10 with a special outdoor cover. Fig. 4, Fig. 4 show the comparison of temperature and humidity measurements of a full day carried out in the city of Bogotá D.C, Colombia. Bogotá has cold weather and is located approximately 2600 meters above sea level. The Fig. 4, Fig. 4, present the magnitude of absolute error of each assembly compared to the reference. It is clear that the error of the RAW sensor is much higher for the two systems with the experimental radiation shields, and increases significantly due to solar radiation. On a smaller scale, it can be seen that the error due to PLA is slightly higher than that from the radiation shield printed in ASA. These observations are consistent with the material properties. Although the results show minimum differences between both materials when experiments first start, the ASA shielded sensors provides results that are more similar to the reference measurements. Due to accelerated outdoor degradation expected of unprotected PLA, the device is expected to get incrementally biased.

**Fig. 5:**
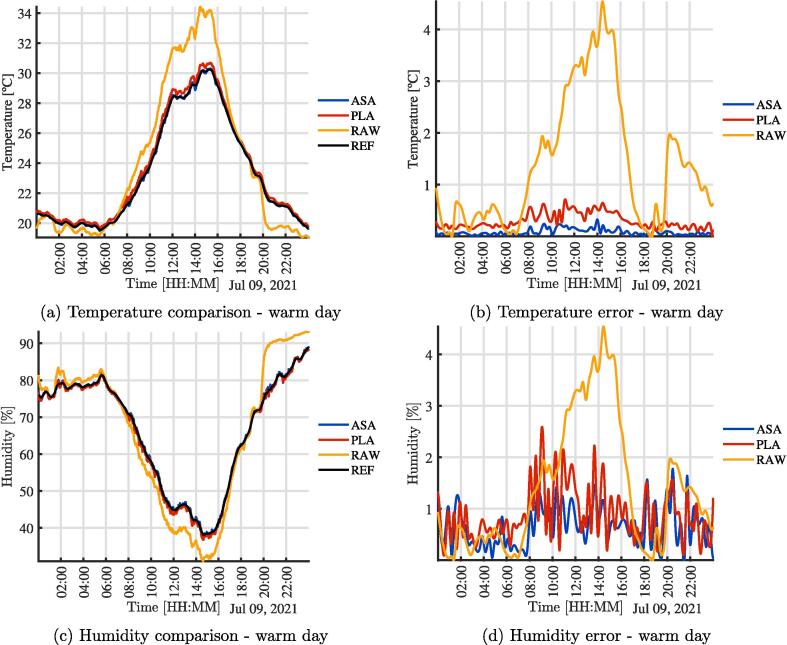
Comparison of temperature and humidity, Medellín, day 2, warm day.

In Fig. 5, the same comparison of the previous experiment is shown, but the experiment was conducted in the city of Medellín, Colombia on a warm day. Medellín is located approximately 1400 meters above sea level. The climate of the city that day was predominantly warm, as can be seen with the temperature values in Fig. 5. Again the impact of the radiation shields can be seen compared to to the effect of radiation on the RAW sensor.

**Fig. 6:**
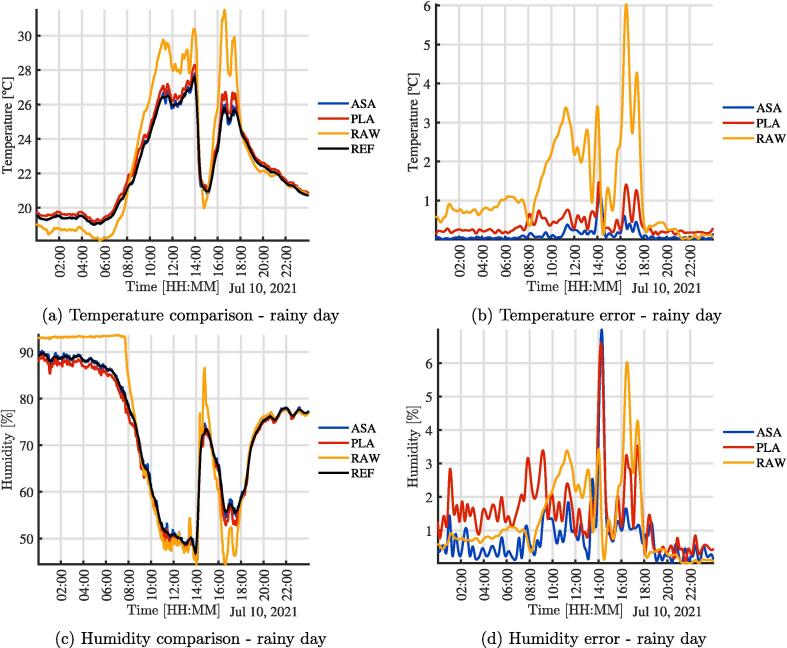
Comparison of temperature and humidity, Medellín, day 3, rainy day.

Finally, in Fig. 6, the comparison for the four sensors is shown in the city of Medellín, during a rainy morning. The effect of precipitation on the RAW sensor are clear. Additionally, it can be seen that as the sensor becomes wet, the measurement of moisture becomes saturated and only approached the reference after drying.

Table 1 and Table 2 show a summary of the maximum, mean and minimum value for the variables and the days, as well as the maximum, mean and minimum error in order to quantify the effectiveness of the radiation shields. It is clear that the ASA error is less than that of the PLA error. The RAW error is much higher than both shielded sensors.

**Table 1:** Limits and error in experimental measurements - Temperature.

	Day 1 - Bogotá			Day 2 - Medellín			Day 3 - Medellín		
	Min	Mean	Max	Min	Mean	Max	Min	Mean	Max
$T_{\mathrm{ASA}}$	12.3900	14.1776	17.3800	19.4619	23.3435	19.0100	19.0100	22.2485	27.7992
$T_{\mathrm{PLA}}$	12.5968	14.3456	17.5159	19.6435	23.5981	30.6800	19.1835	22.5461	28.2988
$T_{\mathrm{RAW}}$	12.3608	14.8831	20.8678	19.0391	24.0499	34.4200	18.1000	22.8252	31.4905
${\mathrm{ET}}_{\mathrm{ASA}}$	00.0032	00.0504	00.4414	00.0104	00.0425	00.3239	00.0010	00.0660	01.1794
${\mathrm{ET}}_{\mathrm{PLA}}$	00.0290	00.1794	00.5129	00.0087	00.2521	00.6635	00.1295	00.3091	01.4731
${\mathrm{ET}}_{\mathrm{RAW}}$	00.0098	00.7275	04.0775	00.0002	01.3449	04.5309	00.0191	01.2075	06.0233

**Table 2:** Limits and error in experimental measurements - Humidity

	Day 1 - Bogotá			Day 2 - Medellín			Day 3 - Medellín		
	Min	Mean	Max	Min	Mean	Max	Min	Mean	Max
$H_{\mathrm{ASA}}$	55.8619	68.6932	80.0026	37.2683	66.2408	88.9683	45.2188	72.4850	90.2487
$H_{\mathrm{PLA}}$	55.9548	68.4776	79.4675	36.7503	65.7930	88.4066	44.6303	71.5258	89.2274
$H_{\mathrm{RAW}}$	47.3603	66.3408	79.5123	30.9499	66.0918	93.1150	44.4492	73.8120	93.6751
${\mathrm{EH}}_{\mathrm{ASA}}$	00.0533	00.3014	03.4173	00.0001	00.3196	01.7675	00.0213	00.3916	06.9984
${\mathrm{EH}}_{\mathrm{PLA}}$	00.0566	00.4020	03.7008	00.0909	00.5152	02.2265	00.0480	01.0521	06.5934
${\mathrm{EH}}_{\mathrm{RAW}}$	00.1200	02.3942	10.8066	00.0939	04.1355	12.6509	00.0760	03.4800	14.3261

**Fig. 7:**
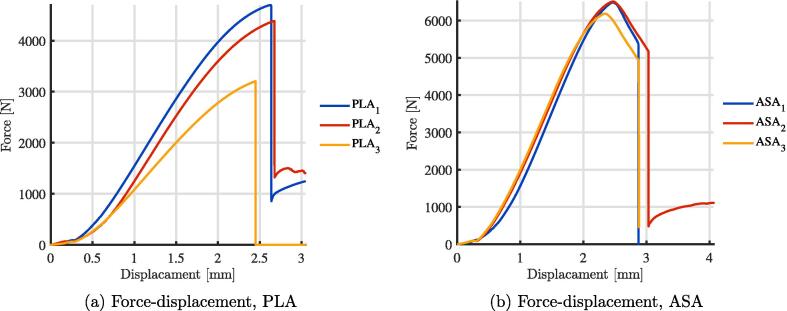
Compression tests after exposure to various environmental conditions.

**Fig. 8:**
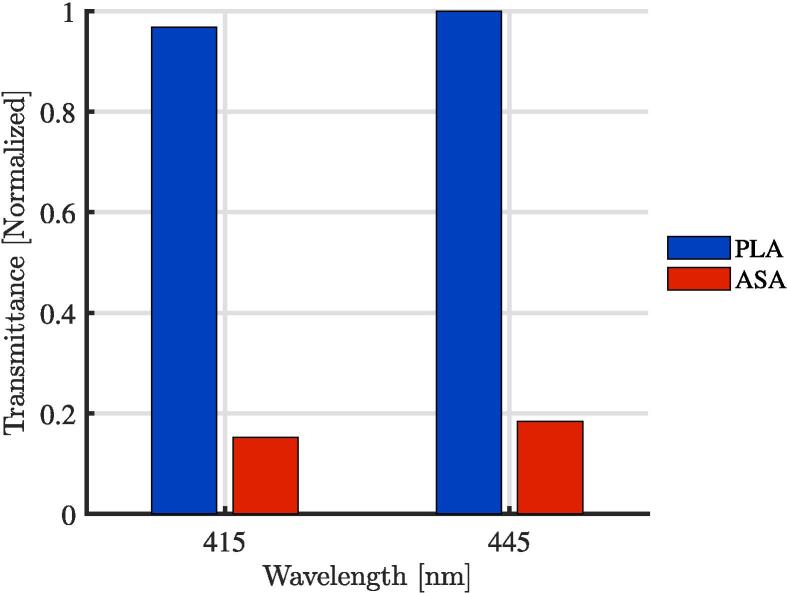
Multispectral sensor-based transmittance test.

**Fig. 9:**
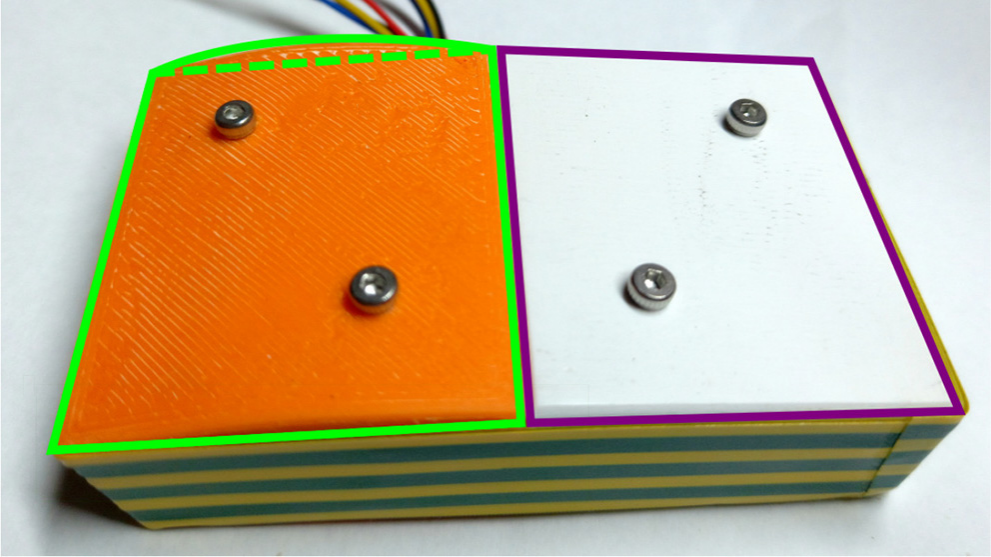
Heat deformation in PLA during transmittance test.

Although PLA and ASA have similar chemical compatibility [32] the primary mode of degradation in this application is from UV radiation reducing the fracture toughness of the 3-D printed parts. To validate this, three ASA and three PLA top plates were printed (part 3 in the Fig. 1) from the same material spool, and with the same infill parameters. One of the plates of each was stored. The second plate was installed in the system and exposed to environmental conditions (UV, heat fluctuations and rain) for 30 days. The final plate underwent the same outdoor testing for 90 days. The stored pieces are wrapped in a protective plastic film and kept in a dry and closed room without natural light where other 3-D printing materials are kept. After being exposed to the various environmental conditions, compression tests were carried out with a universal testing machine at a speed of 5 mm/min on each of the plates. In Fig. 7a, it can see the fracture results for PLA, where ${\mathrm{PLA}}_{1}$ is the part stored, ${\mathrm{PLA}}_{2}$ the part exposed for 30 days and ${\mathrm{PLA}}_{3}$ is the part exposed for 90 days. The same conditions can be seen in the Fig. 7b, for ${\mathrm{ASA}}_{1},{\mathrm{ASA}}_{2}$ and ${\mathrm{ASA}}_{3}$, respectively. It can be seen that the mechanical resistance of both pieces decreased, but that ASA decreased less and the mechanical resistance of the ASA is also higher than that of the PLA. Finally, in Fig. 8, the result of a simple transmittance test is shown. This test was performed by printing two pieces of ASA and PLA with a 2 mm thickness to cover two AS7341 multispectral sensors. The mean transmittance was normalized taking the data of one day. I can be seen that the transmittance of the ASA is less than 20% of that of the PLA for the wavelengths of 415 and 445 nm, which corresponds to the bands close to UV. Finally, Fig. 9 shows the 3-D printed parts for the transmittance test (PLA is orange PLA and ASA is white, as mentioned. Inside this assembly is an AS7341 multispectral sensor. Even after a one day test, the PLA coating deformed significantly and heat and UV deformation is not perceived in the ASA. To observe the deformation in the PLA piece in Fig. 9, the edge was highlighted in green, the deformation in the center reaches 3 mm. On the other hand, the edge of ASA is highlighted with purple, and it can be seen that the deformation is not evident. There are several ways this system can be improved upon in the future. The deformation of the PLA is concerning, but the transmittance and UV stability may be able to be improved with UV protective coatings such as paint or epoxy that could be explored in future work. ASA, however, appears to be a much better candidate material for this application although it is a more expensive filament material. To lower costs, recycled filament or waste plastic particles could be used. Previous work in distributed recycling and additive manufacturing (DRAM) [33] have shown that both PLA and ASA [34] can be recycled into filament as well as direct extrusion printing from particles [35], [36]. In addition, the costs and potentially the longevity can be further enhanced by fabricating the radiation shields with ASA composites [37]. Many other 3-D printing polymers and coatings could be tested for this application in future work, although ASA is the recommended material from this study. Although, ASA is currently a relatively uncommon speciality polymer the costs of this system at nine dollars is still far more cost effective than the several hundred dollars for commercial systems from science suppliers and even the tens of dollars from low-cost vendors. This reduced cost makes the frugal technology more accessible to researchers all over the world [19]. These results are also in agreement with past work that has shown lower costs for the use of 3-D printing in scientific instrumentation in other fields [38], [39], [40]. Future work could match the form factor of the majority of sensors and help scale the radiations shield appropriately for each one. Finally, in the long term future work could focus on integrating and 3-D printing the sensors themselves into the shields[41].

## Human and animal rights

No human or animal studies were conducted in this work.

## Declaration of Competing Interest

The authors declare that they have no known competing financial interests or personal relationships that could have appeared to influence the work reported in this paper.
